# Identifying the Epileptogenic Zone With the Relative Strength of High-Frequency Oscillation: A Stereoelectroencephalography Study

**DOI:** 10.3389/fnhum.2020.00186

**Published:** 2020-06-09

**Authors:** Lei Qi, Xing Fan, Xiaorong Tao, Qi Chai, Kai Zhang, Fangang Meng, Wenhan Hu, Lin Sang, Xiaoli Yang, Hui Qiao

**Affiliations:** ^1^Beijing Neurosurgical Institute, Capital Medical University, Beijing, China; ^2^Beijing Fengtai Hospital, Beijing, China; ^3^Department of Neurosurgery, Beijing Tiantan Hospital, Capital Medical University, Beijing, China

**Keywords:** high-frequency oscillation, wavelet analysis, stereo-EEG, seizure, epilepsy surgery

## Abstract

**Background:**

High-frequency oscillation (HFO) represents a promising biomarker of epileptogenicity. However, the significant interindividual differences among patients limit its application in clinical practice. Here, we applied and evaluated an individualized, frequency-based approach of HFO analysis in stereoelectroencephalography (SEEG) data for localizing the epileptogenic zones (EZs).

**Methods:**

Clinical and SEEG data of 19 patients with drug-resistant focal epilepsy were retrospectively analyzed. The individualized spectral power of all signals recorded by electrode array, i.e., the relative strength of HFO, was computed with a wavelet method for each patient. Subsequently, the clinical value of the relative strength of HFO for identifying the EZ was evaluated.

**Results:**

Focal increase in the relative strength of HFO in SEEG recordings were identified in all 19 patients. HFOs identified inside the clinically identified seizure onset zone had more spectral power than those identified outside (*p* < 0.001), and HFOs in 250–500 Hz band (fast ripples) seemed to be more specific identifying the EZ than in those in 80–250 Hz band (ripples) (*p* < 0.01). The resection of brain regions generating HFOs resulted in a favorable seizure outcome in 17 patients (17/19; 89.5%), while in the cases of other patients with poor outcomes, the brain regions generating HFOs were not removed completely.

**Conclusion:**

The relative strength of HFO, especially fast ripples, is a promising effective biomarker for identifying the EZ and can lead to a favorable seizure outcome if used to guide epilepsy surgery.

## Introduction

Fifty million people all over the world live with epilepsy, and 30% of epileptics suffer from drug-resistant epilepsy, which is defined as a failure to achieve seizure-free status by applying two (or more) appropriately chosen and administered antiepileptic drugs (Kwan et al., 2011; Kalilani et al., 2018). Currently, the optimal treatment for drug-resistant epilepsy is surgical intervention, and the outcome of epilepsy surgery depends greatly on the localization of the brain region that is indispensable for initiating seizures, i.e., the epileptogenic zone (EZ) (Timoney and Rutka, 2017; Jehi, 2018).

The understanding of the EZ is evolving over time. In recent decades, epilepsy has been increasingly considered as a result of brain network disorder, and the concept of the EZ has expanded to “any region of the epileptogenic network,” which opens a door for sophisticated neuroimaging and electrophysiological analyses (Spencer, 2002). To date, various techniques have been applied for investigating the epileptogenic network and identifying the EZ (Jehi, 2018; Nissen et al., 2018). Among those techniques, stereoelectroencephalography (SEEG) has been considered as the current “gold standard” for identifying the EZ by localizing the seizure onset zone in three dimensions (Gonzalez-Martinez, 2016; Bartolomei et al., 2017; Yang et al., 2018). Nevertheless, this “gold standard” still has much room for improvement. SEEG cannot detect seizure spikes efficiently all the time, and the conventional hallmark of epileptic activity, including spikes, is not a specific biomarker for the EZ (Rektor et al., 2009). Accordingly, simply removing the seizure onset zone identified at the time of surgery is not always sufficient to achieve a lasting good surgical outcome (Ramantani et al., 2018). It is necessary to find new specific biomarkers for the EZ to improve the outcome of epilepsy surgery.

Previous studies have verified that in seizures, preictal and interictal discharges show unique patterns on SEEG, and those patterns can be analyzed by specific signal analysis modalities, such as functional connectivity analysis and high-frequency oscillation (HFO) analysis (Bartolomei et al., 2017). HFO, which is defined as at least four oscillations with a frequency over 80 Hz, is suggested to be a promising biomarker of the EZ (Frauscher et al., 2017; Thomschewski et al., 2019). HFO can be divided into ripples (80–250 Hz), fast ripples (250–500 Hz), and very fast ripples (>500 Hz). According to recent studies, all three kinds of HFO have the potential to become valuable biomarkers for identifying the EZ (Brazdil et al., 2017; Hussain et al., 2017; Wang et al., 2017). However, significant interindividual differences are observed in HFO among patients, which prevent its widespread use in clinical practice (Xiang et al., 2009; Gliske et al., 2018). Theoretically, individually normalized HFO may do better in localizing the EZ than raw HFO (Xiang et al., 2009; Gliske et al., 2018).

The current study aimed to refine the HFO-based identification of the EZ by normalizing HFOs in SEEG recordings of each patient. An individualized, frequency-based approach for HFO normalization was applied. The normalization was accomplished by obtaining the relative strength (i.e., the relative spectral power) of each SEEG contact, which could be calculated by dividing the power of each contact by the averaged power of all the contacts. Subsequently, the correlation of the EZ identified by normalized HFOs with surgical outcome was discussed.

## Materials and Methods

### Patients

Nineteen patients with drug-resistant focal epilepsy who underwent SEEG at the Department of Neurosurgery, Beijing Tiantan Hospital between January 2015 and December 2016 were retrospectively analyzed (mean age, 21.2; range, 3–33). The inclusion criteria were patients who underwent comprehensive presurgical non-invasive evaluations including seizure semiology analysis, scalp electroencephalography (EEG), magnetic resonance imaging (MRI), and positron emission computed tomography (PET-CT), and the placement of electrodes was confirmed by postimplantation CT. The exclusion criteria were as follows: (1) no clinical seizure was captured during SEEG monitoring; (2) the seizure onset zone remained undetermined after SEEG monitoring; and (3) the patient received either hemispherectomy or non-resective epilepsy surgery. The current study was approved by the institutional review board of Beijing Tiantan Hospital, and writing informed consents were obtained from all patients or their relatives.

### SEEG Recording and Identification of the Seizure Onset Zone

**FIGURE 1:**
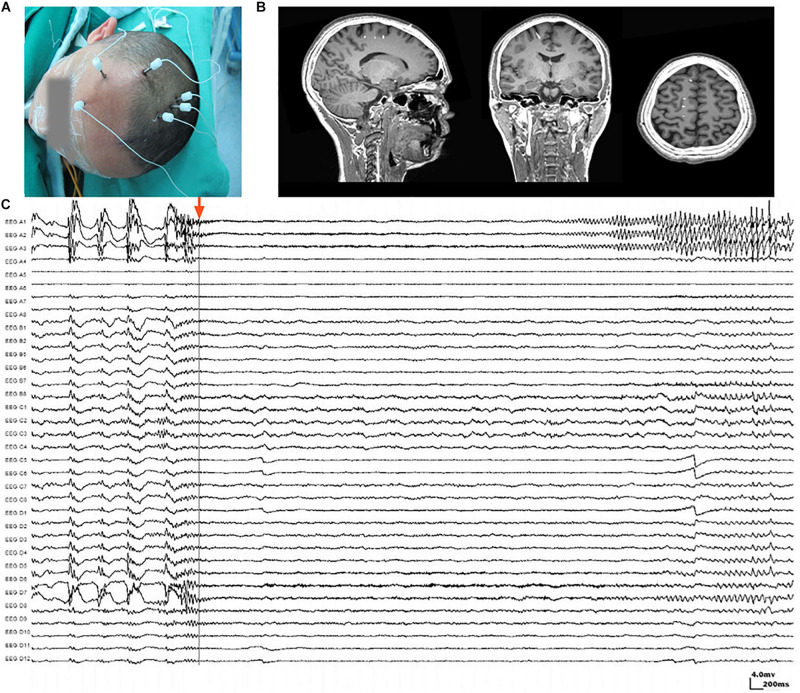
Stereoelectroencephalography (SEEG) electrodes and recordings. **(A)** SEEG electrodes were implanted in a patient in the operating room. **(B)** Localization of SEEG electrode implantation was verified by coregistering preimplantation MRI images with postimplantation CT images. **(C)** A typical SEEG recording of one habitual seizure in patient W, visualized with a 1–100 Hz bandpass, 50 Hz notch filter, at 10 s/page, showing the low voltage fast (LVF) seizure onset type. Channels A1, A2, and A3, which first exhibited low-voltage fast activity, evolving into rhythmic spikes, are recognized as seizure onset zone.

The placement of SEEG depth electrodes (8–16 contacts for each electrode, 0.8 mm diameter, 3.5 mm intercontact distance) was performed by the same surgery team using the Integra CRW System (NeuroSight Arc 2.7.1, Integra Lifesciences Corporation, Plainsboro, NJ, United States). SEEG trajectory planning was done based on routine non-invasive evaluations including semeiology analysis, scalp EEG, MRI, and PET-CT (Figure 1A), and the accuracy of SEEG placement was confirmed by fusing postimplantation CT images with preimplantation MRI images (Figure 1B). SEEG data were recorded with a sampling rate of 2,000 Hz by a 256-channel NK EEG 2100 system (Nihon-Kohden, Tokyo, Japan). The duration of SEEG recording varied individually from 2 to 30 days to ensure that at least two seizures can be captured in each patient. The maximum number of seizures captured in a single patient was 18.

Ictal SEEG recordings of all seizures recorded in each patient were reviewed. The seizure onset zone was identified by at least two experienced epileptologists basing on the channels which recorded earliest rhythmic or semi rhythmic discharges (Singh et al., 2015). Conventional epileptic spikes and slow activities (1–100 Hz, 10 s/page) in SEEG data were used for the presurgical localization of the seizure onset zone, while HFOs were analyzed only for research purpose (Figure 1C). The planned extent of resection was determined by synthesizing seizure semiology and neuroimaging findings.

### SEEG Data Preprocessing

Stereoelectroencephalography data were visually inspected and cleaned from artifacts and noises. Channels with persistent artifacts due to poor contact, environmental or line noise, movement, or others that might affect the results were excluded. Artifact-free epochs, which were at least 2 h away from a seizure, were selected for analyzing interictal activities, while 2-min epochs starting from the detected seizure onset were selected for analyzing ictal activities. Seizure onset was judged by a combination of video recordings and typical SEEG patterns, such as spikes and sharp waves.

### HFO Identification, Quantification, and Normalization

Electroencephalography Studio software (MEG Center, Cincinnati Children’s Hospital Medical Center, Cincinnati, OH, United States) was used to semiautomatically check ripples (80–250 Hz) and fast ripples (250–500 Hz) by searching for increased activities in the corresponding bands (Xiang et al., 2014, 2015b). Subsequently, time–frequency analysis using Morlet continuous wavelet transform was performed for ripples and fast ripples separately to precisely measure the frequency and spectral signals (Xiang et al., 2014, 2015b). The analysis was applied to at least two seizures captured in each patient. For patients who experienced more than one type of seizure, the aforementioned procedures were performed for each type. Coregistered postimplantation CT and preimplantation MRI were used to determine the position of each electrode contact. The waveforms and spectrograms from each channel were then correlated to the corresponding position in structural images for analysis (Figure 1).

To quantify the HFOs generated by a brain region, we computed the spectral power of HFOs with band-pass filtered SEEG data with the conventional approach. To minimize the interindividual variations in baselines and amplitudes, we applied an individualized approach to estimate epileptogenic regions by computing “the relative strength” of HFOs. Mathematically, we divided the spectral power of SEEG signals from each contact by the averaged power of all the contacts on all the electrodes over the entire “clean” segments. The SEEG data processed by this procedure was considered to be spatiotemporally normalized.

### Identification of Brain Regions Generating Epileptic HFOs

The means and standard deviations (SD) of the relative strength of HFOs were computed. The threshold for differentiating pathological HFOs from physiological HFOs was defined as mean plus three times SD (Matsumoto et al., 2013), i.e., HFOs with a spectral peak power over the threshold were identified as pathological HFOs. The contacts that recorded pathological HFOs were considered to be pinpointing to the seizure onset zone.

### Assessments of Resection and Surgical Outcome

Postoperative CT scans were obtained for all patients. To clarify the resection boundaries and their spatial relationship with the electrode contacts, the waveforms of all electrode contacts were identified onto the individual structural images. Postoperative CT and preimplantation MRI were coregistered and then compared with fusion images from postimplantation CT and preimplantation MRI by EEG Studio software.

All patients were followed up for at least 6 months (mean ± SD, 10 ± 2.72; range, 6–15) after surgery, and the surgical outcomes were assessed according to International League Against Epilepsy (ILAE) classifications of epilepsy surgery seizure outcome (Wieser et al., 2001; Durnford et al., 2011). Patients with ILAE classes 1–3 were considered to have a favorable outcome, while those with ILAE classes 4–6 were considered to have a poor outcome.

### Statistical Analysis

Measurements were statistically analyzed with pair-wise comparisons (Student’s *t* test) and multiple analysis of variance (ANOVA). The ratio of activities in brain regions among the electrodes was analyzed by Fisher’s exact test. For multiple comparison, a Bonferroni correction was applied, e.g., since two frequency bands (ripples and fast ripples) were analyzed, only *p* < 0.025 (0.05/2) was considered significant.

## Results

### Clinical Characteristics

A total of 142 SEEG electrodes including 1,654 contacts were implanted in all 19 patients, and 148 seizure-onset channels were identified by clinical neurophysiologists through a preoperative evaluation which is independent of study. Neuropathological examination of resected tissue samples revealed various types of epilepsy-related pathologies. The clinical and pathological characteristics of each patient are summarized in Table 1. Follow-up was achieved for all 19 patients, and 17 of them achieved a favorable seizure outcome (Table 2).

**TABLE 1:** Clinical characteristics of the enrolled 19 patients.

Patients	Age/sex	Seizure type	MRI	PET-CT (hypo-metabolism)	EEG	Location	Pathology
1	24 years/FM	CFS	–	Temporal_Post_Inf_R	O4 & P4-T6	Temporal	FCD Ia
2	25 years/FM	GTCS	–	Postcentral sulus_R	F4 & Fz-Cz	Frontal	FCD Ia
3	29 years/M	CFS	Left temporal polar FCD	Temporal–parietal_L	F7-T3-T5	Frontal–temporal	FCD Ib
4	28 years/M	GTCS	–	Bitemporal (esp Left)	F3-F7	Temporal	FCD IIa
5	31 years/FM	GTCS + CFS	Frontal–parietal–temporal_L FL	Left temporal–insulae–parietal_Inf lobule	F3-F7	Frontal–temporal–insulae	FCD IIa
6	23 years/M	CFS	Frontal_R and Hippocampus_L	Temporal_L–parietal_Inf lobule	T5-P3	Frontal–temporal	FCD IIIa
7	33 years/FM	CFS	Straight gyrus_L FL	Straight gyrus_L	All channel	Frontal	FCD IIb
8	21 years/M	CFS	Left hippocampus–temporal polar	Temporal_ Mid_L	Left anterior head	Temporal	FCDIIIa
9	24 years/FM	GTCS	–	Temporal_Mid_B	All channels	Precentral suclus_R	FCD
10	29 years/M	CFS	–	–	Frontal-Temporal_B	Frontal–temporal	FCDIIa
11	17 years/FM	GTCS	–	Frontal_Mid_Inf_R	Unclear	Frontal	FCD
12	12 years/M	GTCS	Temporal_Mid_L incomplete resectomy	Left Temporal_Ant-TPO	Temporal_B	Temporal	FCDIIIb
13	3 years/M	CFS	–	Frontal_Mid_L	All channels	Frontal–temporal	FCD IIb
14	29 years/M	GTCS	Left temporal horn atrophy	Temporal_L	F7	Temporal	FCD
15	10 years/M	CFS	Temporal_L	Right frontal–temporal–pariental	F4-F8	Frontal–temporal	FCD IIb
16	10 years/M	CFS	White matter FL	Frontal_L–lateral ventricle_B	F4	Frontal_L–lateral ventricle_B	TSC
17	8 years/M	CFS + GTCS	–	NA	F3-F7	Frontal	FCD Ia
18	16 years/M	CFS	–	Temporal_Mid_R–parietal cortex_L	F4-F8	Frontal	FCD Ia
19	30 years/M	GTCS	Insulae_R FL	Temporal_B	All channels	Insulae	FCD Ia

*CFS, complex focal seizure; GTCS, general tonic–colonic seizure; FL, focal lesion; L, left; R, right; B, bilateral; TPO, temporal–parietal–occipital; FCD, focal cortical dysplasia; TSC, tuberous sclerosis complex.*

**TABLE 2:** Relationship between HFOs, SOZ, and surgical outcomes.

Patients	Electrode count	Channel count	Surgical outcome (ILAE)	Number of channels in SOZ	Interictal					Ictal				
					Number of channels with up-threshold HFOs					Number of channels with up-threshold HFOs				
					R	R in SOZ	FR	FR in SOZ	R&FR	R	R in SOZ	FR	FR in SOZ	R&FR
1	6	72	1	15	6	5	5	3	4	4	2	5	1	2
2	10	114	1	2	1	0	0	0	0	7	1	2	0	2
3	10	146	1	4	1	1	0	0	0	12	2	6	1	5
4	6	68	1	9	0	0	1	0	0	5	2	6	0	2
5	8	80	3	6	4	1	3	1	2	6	0	5	0	5
6	10	110	1	5	3	2	1	1	1	13	2	10	1	10
7	7	76	1	3	2	1	1	1	0	6	1	3	0	2
8	9	124	1	11	3	1	1	0	0	15	8	5	0	4
9	4	36	1	3	0	0	1	1	0	5	0	5	0	5
10	10	118	5	9	12	1	0	0	0	23	4	15	0	13
11	6	84	1	14	5	4	0	0	0	3	1	4	1	3
12	8	86	1	8	1	0	0	0	0	15	2	4	0	4
13	4	46	1	11	1	1	1	1	0	7	5	2	0	2
14	6	64	1	16	3	3	12	5	3	12	8	12	6	10
15	6	54	1	8	0	0	0	0	0	4	0	0	0	0
16	11	136	1	3	0	0	0	0	0	2	0	0	0	0
17	6	50	1	12	2	2	4	0	0	5	3	7	1	2
18	8	102	5	6	0	0	0	0	0	7	3	3	0	2
19	7	88	1	12	2	2	2	2	1	2	0	2	0	2

*R, ripples; FR, fast ripples; SOZ, seizure onset zone; HFOs, high-frequency oscillations. For example, in patient 1: the number 6 under R of interictal means that these six channels were detected up-threshold ripples. In these six channels detected ripples, there are five in SOZ. This also applies to FR.*

### The Characteristics of HFOs

**FIGURE 2:**
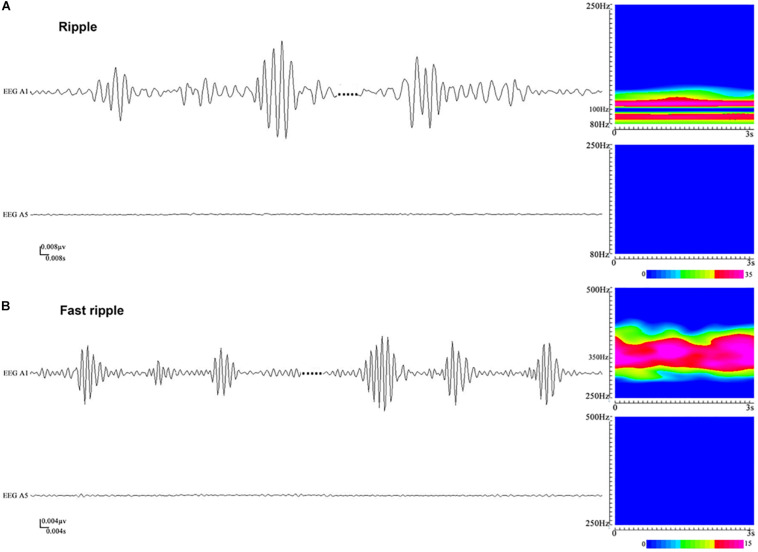
An illustration of high-frequency oscillations (HFOs) in stereoelectroencephalography (SEEG) recordings. **(A)** Ripples in waveforms (80–250 Hz bandpass, 50 Hz notch filter, 0.008 μV/mm, 0.008 s/mm) and accumulated spectrograms. Channel A1, placed inside the seizure onset zone, shows ripples occurring discontinuously, with the frequencies in the range between 88 and 110 Hz. No ripples can be observed in channel A5, which is placed outside the seizure onset zone. **(B)** Fast ripples in waveforms (250–500 Hz bandpass, 50 Hz notch filter, 0.004 μV/mm, 0.004 s/mm) and accumulated spectrograms. Channel A1, placed inside the seizure onset zone, shows fast ripples with the frequencies centered at ∼350 Hz. No fast ripples can be observed in channel A5, placed outside the seizure onset zone.

Artifacts and/or noises were observed in 27 out of 1,654 contacts (1.6%), and the data from these 27 contacts were excluded to ensure the quality of subsequent analysis. Ultimately, SEEG data from 1,627 contacts, including 63 ictal segments (data recorded during seizures), were analyzed. Examples of the identified HFOs are shown in Figure 2. Visual inspection of SEEG data revealed that the amplitude and rate of ripples and fast ripples varied from patient to patient during interictal periods. Even within the same patient, the amplitudes and rates of ripples and fast ripples could vary from time to time.

High-frequency oscillations were identified in all patients. Continuous ripples during ictal periods were observed in 10 of them (15 channels), while continuous fast ripples during ictal periods were only observed in two patients (three channels). In the 10 patients with continuous ripples, 14 out of the total 15 channels were located in the seizure onset zone, which were identified by presurgical evaluation. HFOs were observed both inside and outside the seizure onset zone in each patient.

**FIGURE 3:**
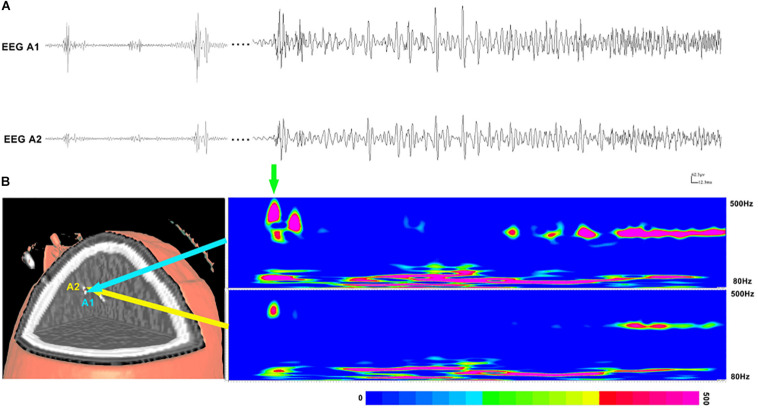
Interictal and ictal HFOs inside the seizure onset zone of two neighboring electrodes. **(A)** High-frequency oscillations (HFOs) (bandpass filtered at 80–500 Hz) in waveforms occur intermittently in interictal periods and occur continuously in the ictal period. **(B)** Time–frequency analysis reveals that the increases in ripples and fast ripples start before ictal onset (the green arrow point). The corresponding sites of the electrodes are shown on the left.

Subsequently, time–frequency analysis was performed. For HFOs inside the seizure onset zone, the spectral power of HFO during ictal periods was significantly increased than that during interictal periods. Figure 3 shows an example of identified HFOs from the interictal to the ictal state. Moreover, we also found that the frequency of ripples increased from interictal to ictal periods: the main frequency range during interictal periods was 85–100 Hz, whereas during the interictal–ictal transition, the main frequency range was 105–130 Hz, accompanied by 85–100 Hz. As for fast ripples, the main frequency range was stable at 250–380 Hz, no significant change was identified.

### Relationship Between the Seizure Onset Zone and HFOs

**FIGURE 4:**
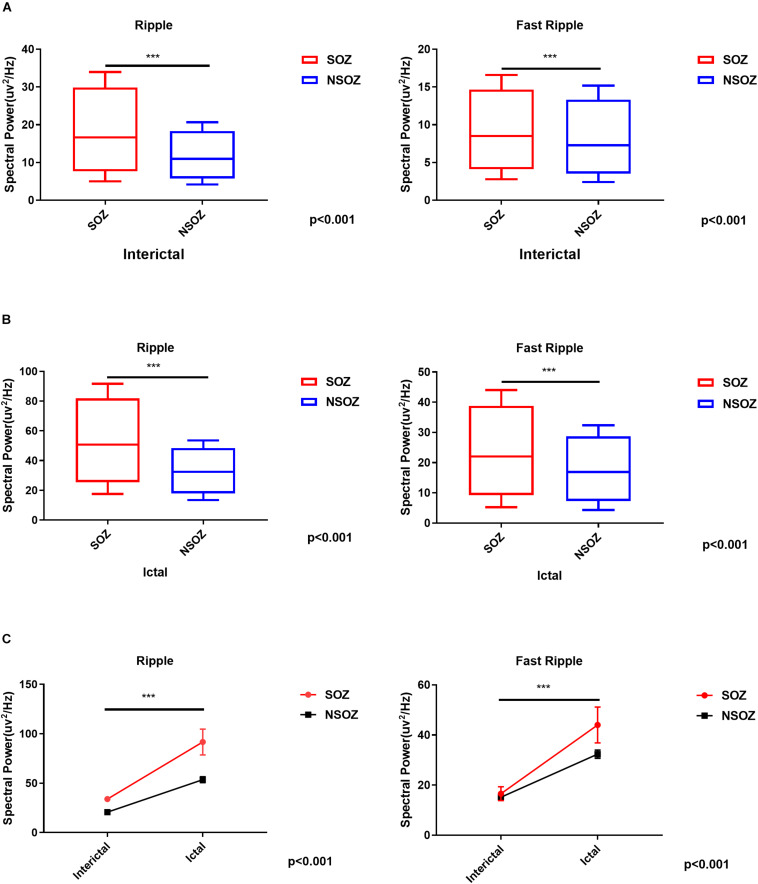
Comparisons of the mean ripple and fast ripple spectral power between the seizure onset zone and non-seizure onset zone during **(A)** interictal and **(B)** ictal periods. **(C)** Mixed-design analyses of variance show dynamic increases of mean ripple and fast ripple spectral power from interictal to ictal periods between the seizure onset zone and non-seizure onset zone. ***p < 0.001.

The spectral data of ripples and fast ripples inside the conventionally defined seizure onset zone (by typical SEEG patterns like spikes and slow activities) showed significantly higher power values than those outside the seizure onset zone (*p* < 0.01). The spectral power for ripples were as follows: (1) interictal, 15.74 ± 7.12 μV^2^/Hz vs. 10.54 ± 4.12 μV^2^/Hz (inside vs. outside the seizure onset zone, for the following data as well) and (2) ictal, 49.02 ± 94.30 μV^2^/Hz vs. 31.61 ± 62.65 μV^2^/Hz (*p* < 0.001). The spectral power for fast ripples were as follows: (1) interictal, 8.15 ± 4.60 μV^2^/Hz vs. 6.88 ± 3.39 μV^2^/Hz; and (2) ictal: 21.04 ± 34.32 μV^2^/Hz vs. 16.21 ± 24.26 μV^2^/Hz (*p* < 0.001). Additionally, the spectral data also showed an increase in HFO power from interictal to ictal periods. Figure 4 shows the comparisons of HFO power during interictal and ictal periods and its spectral changes between the seizure onset zone and non-seizure onset zone. The spectrograms revealed an increase in HFO power in 105–130 Hz band (ripple) and 250–380 Hz band (fast ripple) during interictal and ictal periods.

To estimate the EZ, the number of channels with epileptic HFOs were quantified. During interictal periods, ripples were observed in 2.32 ± 2.88 channels, and fast ripples were observed in 1.58 ± 2.83 channels (*p* < 0.01). During ictal periods, the HFO involved regions tended to be larger, with ripple regions refer to 8.00 ± 5.43 channels and fast ripple regions refer to 5.00 ± 3.80 channels. The overlap of the ripple regions inside the seizure onset zone was 0.71 ± 0.32 channel sites for interictal periods and 0.37 ± 0.20 channel sites for ictal periods, and the overlap of the fast ripple regions was 0.79 ± 0.27 channels for interictal periods and 0.23 ± 0.13 channels for ictal periods. Notably, the fast ripples were more spatially focal than the ripples. In addition, the overlap between the identified ripple and fast ripple regions was 0.16 ± 0.29 channels during interictal periods and 0.50 ± 0.31 channels during ictal periods, whereas the inverse overlap was 0.17 ± 0.31 channels during interictal periods and 0.71 ± 0.33 channels during ictal periods, which suggested that ripples and fast ripples were independent events in the epileptic brain.

### Surgical Outcome and HFO Regions

Overall, 16 patients achieved seizure freedom and were classified as ILAE class 1. One patient experienced a seizure after surgery, which was different from his habitual seizures, and was classified as ILAE class 3. Two patients experienced frequent seizures postoperatively and were classified as ILAE class 5.

For the 17 (89.5%) patients with a favorable outcome, the proportion of the resected areas in ripple-generating brain regions (ripple resection rate) was 0.76 ± 0.41 for interictal periods and 0.38 ± 0.34 for ictal periods. The identified fast ripple resection rate was higher than the ripple resection rate in interictal periods (0.91 ± 0.16), but lower in ictal periods (0.33 ± 0.33). Notably, ripples and fast ripples were propagated after seizure onset, especially to the symptomatic regions. In addition, we reviewed interictal HFOs for all 17 patients with a favorable outcome and found that the region presenting the highest spectral values for fast ripples had been resected; however, for the two patients with poor outcomes, the brain regions generating fast ripples had not been removed completely. This result indicated that the resection of brain regions generating fast ripples was critical for the achievement of a favorable seizure outcome.

## Discussion

In the current study, we analyzed SEEG data using an individualized, frequency-based approach for HFO analysis and demonstrated that the resection of the EZ estimated by the relative strength of HFOs was associated with a favorable seizure outcome. Notably, HFOs in 250–500 Hz were more specific for localizing the EZ. The results enhanced the clinical application value of HFO analysis in patients with drug-resistant epilepsy and provided a potential marker for guiding epilepsy surgery.

The detection of HFOs has been widely studied in intracranial EEG studies but rarely in SEEG studies (Chassoux, 2003; Amiri et al., 2016; Ferrari-Marinho et al., 2016; Fuertinger et al., 2016). Although both subdural and stereotactic electrode recordings can be used to identify the EZ, they exhibit some differences. The electrodes used for SEEG are thin, needle-like electrodes, which can be implanted stereotactically, i.e., with the aid of a stereotactic frame using precalculated coordinates that guarantee accurate targeting of specific deep regions in the brain. Currently, SEEG has been indicated as a preferable tool for the epileptogenic network exploration and is especially effective in detecting the epileptogenic activity originate from deep brain structures. Furthermore, previous reports have shown that a high rate of HFOs can be used for the localization of the EZ (Chaitanya et al., 2015; Ferrari-Marinho et al., 2016; Jacobs et al., 2016). Accordingly, a combination of SEEG recording and HFO analysis is considered to be a promising approach for identifying the EZ.

Generally, HFOs can be identified through visual inspection of bandpass-filtered data. However, this process is quite time consuming and highly dependent on the reviewers. For instance, the identification of HFO necessitates at least four consecutive oscillations above the “background or baseline,” and the “baseline” is generally determined by reviewers subjectively. Additionally, previous studies showed that the characteristics of HFOs in epileptic brain varies greatly among individuals (Staba et al., 2014; Chaitanya et al., 2015; Ferrari-Marinho et al., 2016). As observed in the current study, the morphology, amplitude, and duration of HFOs varied not only among different individuals but also among time to time, site to site in the same patient. All these factors present significant barriers to the clinical application of HFO analysis for the localization of the EZ.

In the current study, we improved the method for HFO analysis. With wavelet transforms, the spectrograms revealed that the spectral power of ripples was significantly increased in 105–130 Hz band, while the spectral power of fast ripples was relatively stable at 250–380 Hz band. To minimize interindividual variation, we computed the relative strength of HFOs of each electrode contact, and the EZ identified by the relative strength of HFOs and the seizure onset zone defined by preoperative evaluation were consistently matched. Importantly, the resection of brain regions with high relative strength of HFOs was correlated with a favorable surgical outcome. According to previous reports, epilepsy surgery, which is performed typically based on the seizure onset zone defined by spike-and-wave discharges (low-frequency signals), conventionally achieve seizure freedom in 40–70% of patients with medically refractory focal epilepsy (Engel et al., 2003). In the current study, seizure freedom was achieved in 89.5% of patients, and the brain regions with high relative strength of HFOs were removed completely in all of them, while the remaining two patients with poor outcomes showed residual brain regions generating HFOs after surgery. The results suggested that the relative strength of HFOs is an effective biomarker for identifying the EZ and can be used for the presurgical evaluation of patients with drug-resistant epilepsy. Compared with visual inspection, this approach is individually normalized, objective, quantitative, and time efficient (semiautomatically computed by a computer). Moreover, it provides a precise frequency description of epileptic activities.

It has been shown that HFOs can reliably localize the EZ in both mesial temporal and extratemporal epilepsy (Jacobs et al., 2009; Cho et al., 2014). In our study, the combination of postsurgical CT and spectrograms revealed that, compared with the EZ identified by the conventional spikes in routine EEG analysis, the EZ identified by HFO analysis was more focal, which was consistent with previous reports (Xiang et al., 2014, 2015a). Additionally, our analysis showed that fast ripples (250–500 Hz) were generally more focal than ripples (80–250 Hz), which indicated that fast ripples may have greater clinical value than ripples for identifying the EZ. The other relevant finding was that the changes in ripples and fast ripples were not always spatially overlapped, indicating that the ripples and fast ripples are mutually independent biomarkers for identifying the EZ.

The results of the present study also demonstrated the dynamic changes in HFOs. HFOs were observed to be significantly increased from the interictal to ictal period. Consistent bursts of ripples and fast ripple could be observed during ictal periods, and sudden increases of fast ripples were typically identified inside seizure onset zone. Such dynamic changes in HFOs inside the seizure onset zone could be used to indicate the interictal–ictal transition. In other words, the dynamic changes in HFOs could be used to predict epileptic state changes. This finding also provide support to the notion that HFOs represent the network-driven activity of the brain in epilepsy.

The current investigation was limited by its retrospective nature. Moreover, SEEG electrodes implantation is highly relied on the preimplantation plan, which can greatly affect the positional distribution of the contacts, making presurgical evaluation of the EZ a challenging task. In addition, our work is limited to analyzing the correlation between the identified HFOs and the conventional seizure onset zone, and characterizing the relationship between HFOs and the surgical outcome. Such limitation arises from the fact that there is no current “gold standard” for identifying HFOs (Spring et al., 2017). Overall, the promising results lead us to believe that HFO analysis can be refined to improve the identification of the EZ and will play a key role in the presurgical evaluation of epilepsy surgery in the near future. Further studies are required to determine a reliable, effective quantitative marker of HFO activity for delineating the true EZ.

## Data Availability Statement

The datasets generated for this study are available on request to the corresponding author.

## Ethics Statement

The studies involving human participants were reviewed and approved by the Institutional Review Board of Beijing Tiantan Hospital. Written informed consent to participate in this study was provided by the participants, and where necessary, the legal guardian/next of kin.

## Author Contributions

LQ and XF were the major contributors in data analysis and manuscript writing. KZ, LS, WH, and XY contributed to the diagnosis and treatment of patients. XT and HQ contributed to checking the manuscript. All authors read and approved the manuscript.

## Conflict of Interest

The authors declare that the research was conducted in the absence of any commercial or financial relationships that could be construed as a potential conflict of interest.
